# Gradients of thalamic connectivity in the macaque lateral prefrontal cortex

**DOI:** 10.3389/fnint.2023.1239426

**Published:** 2023-10-16

**Authors:** Elena Borra, Marianna Rizzo, Giuseppe Luppino

**Affiliations:** Neuroscience Unit, Department of Medicine and Surgery, University of Parma, Parma, Italy

**Keywords:** thalamus, thalamocortical, medialis dorsalis, pulvinar, executive functions, parieto-frontal circuits

## Abstract

In the primate brain, the lateral prefrontal cortex (LPF) is a large, heterogeneous region critically involved in the cognitive control of behavior, consisting of several connectionally and functionally distinct areas. Studies in macaques provided evidence for distinctive patterns of cortical connectivity between architectonic areas located at different dorsoventral levels and for rostrocaudal gradients of parietal and frontal connections in the three main architectonic LPF areas: 46d, 46v, and 12r. In the present study, based on tracer injections placed at different dorsoventral and rostrocaudal cortical levels, we have examined the thalamic projections to the LPF to examine to what extent fine-grained connectional gradients of cortical connectivity are reflected in the topography of thalamo-LPF projections. The results showed mapping onto the nucleus medialis dorsalis (MD), by far the major source of thalamic input to the LPF, of rostral-to-caudal LPF zones, in which MD zones projecting to more caudal LPF sectors are located more rostral than those projecting to intermediate LPF sectors. Furthermore, the MD zones projecting to the rostral LPF sectors tended to be much more extensive in the rostrocaudal direction. One rostrolateral MD sector appeared to be a common source of projections to caudal prefrontal areas involved in the oculomotor frontal domain, a more caudal and ventral MD sector to a large extent of the ventral LPF, and middle and dorsal MD sectors to most of the dorsal LPF. Additional topographically organized projections to LPF areas originated from the nucleus pulvinaris medialis and projections from the nucleus anterior medialis selectively targeted more rostral sectors of LPF. Thus, the present data suggest that the topography of the MD-LPF projections does not adhere to simple topological rules, but is mainly organized according to functional criteria.

## 1. Introduction

The lateral prefrontal cortex (LPF) is a large, heterogeneous region involved in the so-called executive functions, i.e., those mechanisms by which behavioral performance is optimized in situations requiring cognitive processes (Tanji and Hoshi, 2008). Non-human primate studies have shown that the LPF hosts connectionally and functionally distinct areas. These areas were originally described in terms of higher-order processing of different aspects of sensory information encoded in working memory (Goldman-Rakic, 1987; Levy and Goldman-Rakic, 2000) and then associated with the control of behavioral planning (see Miller and Cohen, 2001; Tanji and Hoshi, 2008).

Specifically, rostral to the prearcuate frontal oculomotor domain, including areas 8-FEF, 8r, 8A, 45B, and 45A (see Borra and Luppino, 2021), the LPF within and along the principal sulcus (PS) corresponding to area 46 of Walker (1940) can be subdivided into a dorsal part (46d), characterized by connectivity with superior and medial parietal areas, and a ventral part (46v), characterized by connectivity with inferior parietal areas (Tanji and Hoshi, 2008). Dorsal to area 46d, area 9 is characterized by connectivity with superior temporal areas (Petrides and Pandya, 1999), whereas ventral to area 46v, rostral area 12 (12r), as defined by Carmichael and Price (1994), is characterized by connectivity with inferotemporal areas (Webster et al., 1994).

In a series of studies focused on the connectivity of LPF areas, we have provided evidence for rostrocaudal connectional gradients in areas 46d, 46v, and 12r, in which the caudal part is primarily connected with parietal and prearcuate oculomotor areas, the middle part with parietal and frontal skeletomotor areas, and the rostral part primarily with other prefrontal areas (Borra et al., 2011, 2019; Gerbella et al., 2013). Altogether, these data suggest a general rostrocaudal organization of the macaque LPF in which more caudal and intermediate parts of areas 46d, 46v, and 12r are differentially involved in the executive control of oculomotor and skeletomotor behavior, respectively, and more rostral parts are most likely involved in higher-order, possibly more abstract, cognitive functions. This LPF connectional architecture is a potential substrate for models of executive functions in humans based on a rostrocaudal hierarchical organization of cognitive processing with more anterior regions involved in progressively more abstract processing (Koechlin and Summerfield, 2007).

It is well established that the prefrontal cortex, as a whole, has a strong relationship with higher-order thalamic nuclei, especially the medialis dorsalis (MD), but also several others, including the ventralis anterior, pars magnocellularis (VAmc), the pulvinaris medialis (Pul.m), and the anterior medialis (AM; see, e.g., Phillips et al., 2021). Specifically, the MD, which is the major source of projections to the LPF, hosts distinct subdivisions showing distinct patterns of subcortical and cortical connections (see Mitchell, 2015; Phillips et al., 2019, 2021). The projections from MD to LPF, as well as those from other higher-order thalamic nuclei, might have multiple functional roles: (i) relaying specific subcortical outputs to specific cortical sectors or areas; (ii) mediating trans-thalamic information flow between different, distant, or close cortical areas (e.g., Sherman, 2007); (iii) controlling the gain (excitability) and sustaining activity of cortical neurons (Phillips et al., 2021). By virtue of its connectivity with the prefrontal cortex, the MD is considered to contribute to all aspects of cognitive control (Phillips et al., 2021), including regulating the plasticity and flexibility of prefrontal-dependent cognitive functions (Baxter, 2013) and supporting the transfer of information for learning new information and adaptive decision-making (Mitchell, 2015; Perry et al., 2021).

Several studies have examined the topography of the connections between the MD and the prefrontal cortex, and several different connectional models have been proposed (Pribram et al., 1953; Kievit and Kuypers, 1977; Goldman-Rakic and Porrino, 1985; Barbas et al., 1991; Siwek and Pandya, 1991; Erickson and Lewis, 2004; Phillips et al., 2019). These studies, based on cortical lesions, relatively large neural tracer injection sites, or tracer injections in the MD, could not provide fine-grained information on the topography of the MD-prefrontal connections, which is fundamental to further understanding the way in which the MD and the LPF cooperate in cognitive processes.

In the present study, based on tracer injections placed at different rostrocaudal levels in areas 12r, 46v, and 46d, in the caudally adjacent area 8 rostral (8r), and, more dorsal, at different rostrocaudal levels in areas 8B and 9, we have examined the thalamic projections to the LPF to examine to what extent fine-grained connectional gradients of cortical connectivity in the LPF are reflected in the topography of MD-LPF projections. The thalamic connectivity of the more caudal prearcuate areas 8-FEF, 45B, and 45A has been described in a previous study (Contini et al., 2010).

## 2. Methods

### 2.1. Subjects, surgical procedures, and selection of the injection sites

The present study is based on results from injections of neural tracers placed in the LPF areas 12r, 46v, 46d, 8r, 8B, and 9 in 11 macaque monkeys (5 *Macaca fascicularis* and 6 *Macaca mulatta*). Table 1 summarizes the locations of the tracer injections, the injected tracers, and their amounts. Most of these cases have already been used in previous studies focused on the cortical connectivity of the areas under study (Gerbella et al., 2010, 2013; Borra et al., 2011, 2019). Animal handling and surgical and experimental procedures complied with the European law on the humane care and use of laboratory animals (directives 86/609/EEC, 2003/65/CE, and 2010/63/EU) and Italian laws regarding the care and use of laboratory animals (D.L. 116/92 and 26/2014) and were periodically approved by the Veterinarian Animal Care and Use Committee of the University of Parma and authorized by the Italian Ministry of Health.

**Table 1:** Animals used, location of injection sites, and type and amount of injected tracers.

**Case**	**Species**	**Hemisphere**	**Area**	**Tracer**	**Amount (μl)**
39	*M. fascicularis*	R	caudal 12r^a^	DY 2%	1 × 0.2 μl
		R	8r^a^	FB 3%	1 × 0.2 μl
43	*M. mulatta*	L	intermediate 12r^b^	FB 3%	1 × 0.2 μl
		R	caudal 46v^c^	FR 10%	1 × 1 μl
44	*M. mulatta*	L	caudal 46v^c^	DY 2%	1 × 0.2 μl
		L	intermediate 46v^c^	FB 3%	1 × 0.2 μl
		R	intermediate 12r^b^	LYD 10%	1 × 1 μl
48	*M. mulatta*	R	rostral 12r^b^	FB 3%	1 × 0.2 μl
		L	caudal 12r^b^	LYD 10%	1 × 1.3 μl
51	*M. mulatta*	L	intermediate 46v^c^	FB	1 × 0.2 μl
56	*M. mulatta*	L	8r	FR 10%	2 × 1 μl
57	*M. fascicularis*	R	intermediate 46d^d^	WGA 4%	1 × 0.3 μl
58	*M. fascicularis*	R	caudal 46d^d^	FB 3%	1 × 0.2 μl
		L	9	LYD 10%	1 × 1.5 μl
		L	8B	FR 10%	1 × 1 μl
60	*M. fascicularis*	L	intermediate 46d^d^	FB 3%	1 × 0.3 μl
		R	caudal 46d^d^	FR 10%	1 × 1.8 μl
61	*M. mulatta*	R	rostral 46v^c^	DY 2%	2 × 0.2 μl
64	*M. fascicularis*	R	rostral 46d	FB 3%	1 × 0.2 μl
		L	rostral 46d^d^	CTBg 1%	2 × 0.75 μl

Cortical labeling described in

a Gerbella et al. (2010),

b Borra et al. (2011),

c Gerbella et al. (2013), and

d Borra et al. (2019).

Under general anesthesia and aseptic conditions, each animal was placed in a stereotaxic apparatus, and an incision was made on the scalp. The skull was trephined to remove the bone overlying the target region, and the dura was opened to expose the LPF.

**Figure 1:**
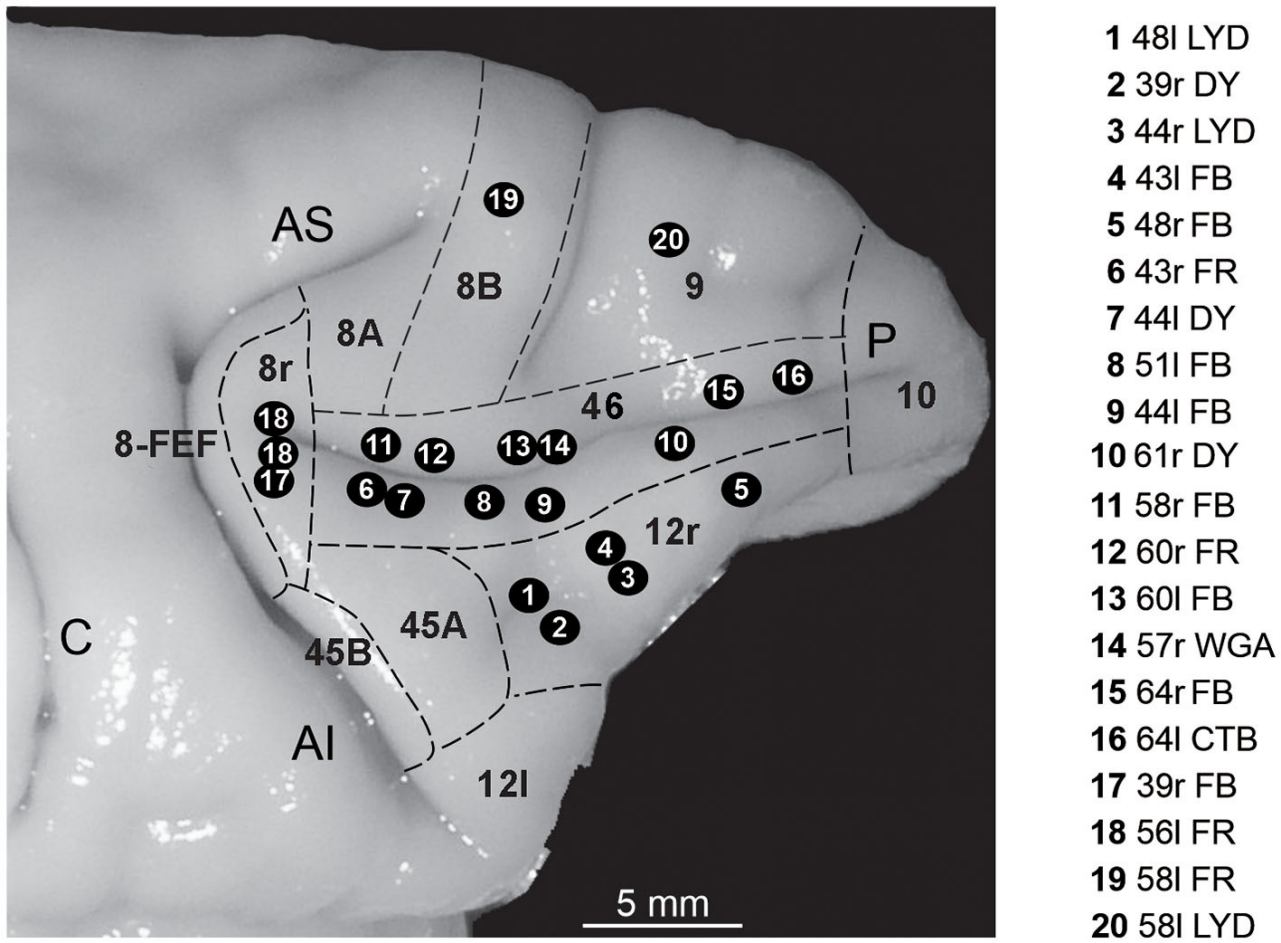
Composite view of all the injection sites used in the present study, mapped on a template hemisphere (Case 44r), shown as numbered black circles. Injection sites in areas 46v and 46d were reported on the template hemisphere based on their distance from the rostral border of area 8r (Gerbella et al., 2013; Borra et al., 2019), and those in area 12r based on their AP position relative to the rostrocaudal extent of the PS (Borra et al., 2011). In Case 56l (number 18 in the figure), there were two distinct FR injection sites. Dashed lines mark the cytoarchitectonic borders of lateral prefrontal areas. AI, inferior arcuate sulcus; AS, superior arcuate sulcus; C, central sulcus; P, principal sulcus.

The choice of the injection sites was based on identified anatomical landmarks of the LPF (superior and inferior arcuate sulcus, principal sulcus, and infraprincipal dimple) and using an average architectonic map providing an estimate of the location of the various areas of the caudal part of the LPF (Gerbella et al., 2007). These data were then used to estimate the location of area 8r, the caudal border of areas 12r and 46v and that between the two areas, and the caudal border of area 46d (Figure 1).

After the tracer injections, the dural flap was sutured, the bone was replaced, and the superficial tissues were sutured in layers. During surgery, hydration was maintained with saline, and temperature was maintained using a heating pad. Heart rate, blood pressure, respiratory depth, and body temperature were continually monitored. Upon recovery from anesthesia, the animals were returned to their home cages and closely monitored. Dexamethasone and prophylactic broad-spectrum antibiotics were administered preoperatively and postoperatively, similar to analgesics.

### 2.2. Tracer injections and histological procedures

Once the appropriate site was chosen, the neural tracers Fast Blue (FB, 3% in distilled water, Dr. Illing Plastics GmbH, Breuberg, Germany), diamidino yellow (DY, 2% in 0.2 M phosphate buffer at pH 7.2, Dr. Illing Plastics), dextran conjugated with Lucifer yellow (LYD; 10,000 MW, 10% 0.1 M phosphate buffer, pH 7.4, Invitrogen, Thermo Fisher Scientific), or with tetramethylrhodamine (Fluoro-Ruby, FR, 10% 0.1 M phosphate buffer, pH 7.4; Invitrogen), wheat germ agglutinin (WGA; 4% in distilled water, Vector Laboratories, Burlingame, CA), and cholera toxin B subunit, conjugated with Alexa488 (CTB green, CTBg, 1% in 0.01 M PBS at pH 7.4, Invitrogen, Thermo Fisher Scientific) were slowly pressure-injected through a glass micropipette (tip diameter: 50–100 μm) attached to a 1- or 5-μl Hamilton microsyringe (Reno, NV, USA) positioned with a stereotaxic holder.

After appropriate survival periods following the injections (28 days for FR and LYD, 12–14 days for FB, DY, and CTBg, and 48 h for WGA), each animal was deeply anesthetized with an overdose of sodium thiopental and perfused through the left cardiac ventricle consecutively with saline (2 L in 10 min), 3.5% formaldehyde (5 L in 30 min), and 5% glycerol (3 L in 20 min), all prepared in 0.1 M phosphate buffer, pH 7.4. Before removing the brain from the skull, the animal was placed in a stereotaxic apparatus, and the brain was blocked along the stereotaxic coronal plane. Each brain was then photographed and placed in 10% buffered glycerol for 3 days and 20% buffered glycerol for 4 days, and finally cut frozen into coronal sections of 60-μm thickness.

In all the cases in which FB and DY were injected, sections spaced 300 μm apart—that is, one section in each repeating series of five—were mounted, air-dried, and quickly coverslipped for epifluorescence microscopy. Other series of sections spaced 300 μm apart were processed for visualizing LYD (Cases 44r, 48l, and 58l), FR (Cases 43r, 56l, and 58l), CTBg (Case 64l), or WGA (Case 57r) with immunohistochemistry. As in all cases, an additional injection of the axonal tracer biotinylated dextran-amine (BDA) was placed in a different part of the cortex; these sections were processed for the visualization of both BDA and FR, LYD, CTBg, or WGA using the double labeling protocol described in detail in Gerbella et al. (2010, 2016).

Briefly, the sections were first processed to visualize BDA, i.e., incubated overnight in the ABC solution (VECTASTAIN ABC kit, PK-4000, Vector Laboratories), and then BDA was stained brown using 3,3′-diaminobenzidine (DAB, Sigma-Aldrich, St. Louis, MO). Then, the sections were incubated overnight in avidin-biotin blocking reagent (SP-2001, Vector Laboratories) and for 72 h at 4°C in a primary antibody solution of rabbit anti-FR, rabbit anti-LY (1:3000; Invitrogen), or rabbit anti-Alexa 488 (1:15 000, Thermo Fisher Scientific) in 0.5% Triton and 5% normal goat serum in PBS, or overnight at room temperature in a primary antibody solution of goat anti-WGA (1:2000; Vector Laboratories) in 0.3% Triton and 5% normal rabbit serum in PBS. The sections were then incubated for 1 h in biotinylated secondary antibody (1:200, Vector Laboratories) in 0.3% Triton and 5% normal goat serum (normal rabbit serum for WGA) in PBS. Finally, FR, LYD, CTBg, and WGA labelings were visualized using the VECTASTAIN ABC kit and the Vector SG peroxidase substrate kit (SK-4700, Vector Laboratories) as a chromogen. With this procedure, BDA labeling was stained brown, and the FR, LYD, WGA, or CTB labeling was stained blue in the same tissue sections.

In all cases, one series of each fifth section was stained using the Nissl method (0.1% thionin in 0.1 M acetate buffer, pH 3.7).

### 2.3. Data analysis

#### 2.3.1. Injection sites and distribution of retrogradely labeled neurons

All the injection sites used in this study were completely restricted to the cortical gray matter; in some cases, they involved the entire cortical thickness, while in others mostly layers III–V. For the areal attribution of the injection sites, the LPF was subdivided as shown in Figure 1, according to cyto- and chemo-architectonic criteria described in Carmichael and Price (1994), Gerbella et al. (2007), and Saleem et al. (2014) and adopted in our previous studies focused on the cortical connectivity of the areas under study (Gerbella et al., 2010, 2013; Borra et al., 2011, 2019).

The distribution of retrogradely labeled thalamic neurons was mapped in sections every 300 μm, together with the outline of the thalamus, ventricles, and blood vessels, using a computer-based charting system. Borders of thalamic nuclei, defined in adjacent Nissl-stained sections, were then superimposed on the plots of labeled cells, using the outline of the thalamus, ventricles, and blood vessels, with the aid of a microprojector and a camera lucida, and, if necessary, correcting differences in shrinkage by slightly changing their magnification.

Data from individual sections were also imported into three-dimensional (3D) reconstruction software (Bettio et al., 2001), providing volumetric reconstructions of the MD, including connectional and architectonic data. The overall labeling distribution in the various MD subdivisions was then visualized in the dorsal and lateral views of the 3D reconstructions of the nucleus and in 500-μm-thick horizontal sections, re-sliced from the 3D reconstructions.

In all cases and for all tracer injections, the relative contribution of the input from different thalamic nuclei to the areas under study was assessed by counting, for each tracer injection, the number of labeled cells in each thalamic nucleus. The absolute number of labeled neurons was largely variable across cases, which may be accounted for by several factors (e.g., differences in amount, spread, and sensitivity of injected tracers). Thus, for each tracer injection, afferents to the injected cortical field were expressed in terms of the percent of labeled neurons found in a given thalamic nucleus with respect to the total number of labeled cells in the thalamus.

#### 2.3.2. Definition of thalamic nuclei

The borders of the thalamic nuclei were primarily defined according to the cytoarchitectonic criteria, nomenclature, and anteroposterior (AP) levels of Olszewski's atlas of the thalamus (Olszewski, 1952), based on stereotaxic coronal sections. Specifically, in the MD, where most of the observed thalamic labeling was located, three main subdivisions were recognized: a magnocellularis (MDmc), a parvicellularis (MDpc), and a multiformis (MDmf) part. MDmc occupies the medial part of the rostral two-thirds of the MD, from approximately AP level 4.5 to approximately AP level 8.4, invading, rostrally, the ventral part. Relatively large, darkly staining, and almost evenly distributed multipolar cells characterize this subdivision. MDpc occupies most of the remaining part of the MD. Its cells tend to be smaller and have paler staining than in MDmc and are variable in size and unevenly distributed. MDmf is located at the lateral edge of the rostral two-thirds of the MD, bordering laterally with the internal medullary lamina. It is characterized by large, darkly staining, spindle-shaped, or multipolar cells lying isolated or in small groups among small, pale staining cells. In the caudal MD, MDmf is replaced by the densocellularis (MDdc) part of the MD, populated by large, darkly staining cells and considered by Jones (1985) part of the intralaminar nuclei. Finally, rostral to approximately AP level 8.7, the rostral pole of the MD displays an almost homogeneous cytoarchitecture and has been designated as rostral MD (MDr).

## 3. Results

As expected, most of the thalamic projections to the LPF areas under study originated from the MD nuclear complex, with additional contributions mostly from the Pul.m and, in some cases, the VAmc or AM nuclei. The contribution of intralaminar nuclei was weak and variable across cases. In the MD nuclear complex, the distribution of the labeling within and among the various subdivisions largely varied qualitatively and quantitatively according to the injected area or sector of the LPF areas under study.

In general, the retrograde labeling observed in the MD was typically organized in close aggregates of labeled cells, approximately 250–500 μm in size, more or less neatly separated by unlabeled zones. Furthermore, in those cases in which retro-anterograde tracers (LYD or FR) were injected, the anterograde labeling overlapped with the retrograde one but tended to be more extensive and more evenly distributed.

### 3.1. Thalamic projections to area 12r

**Figure 2:**
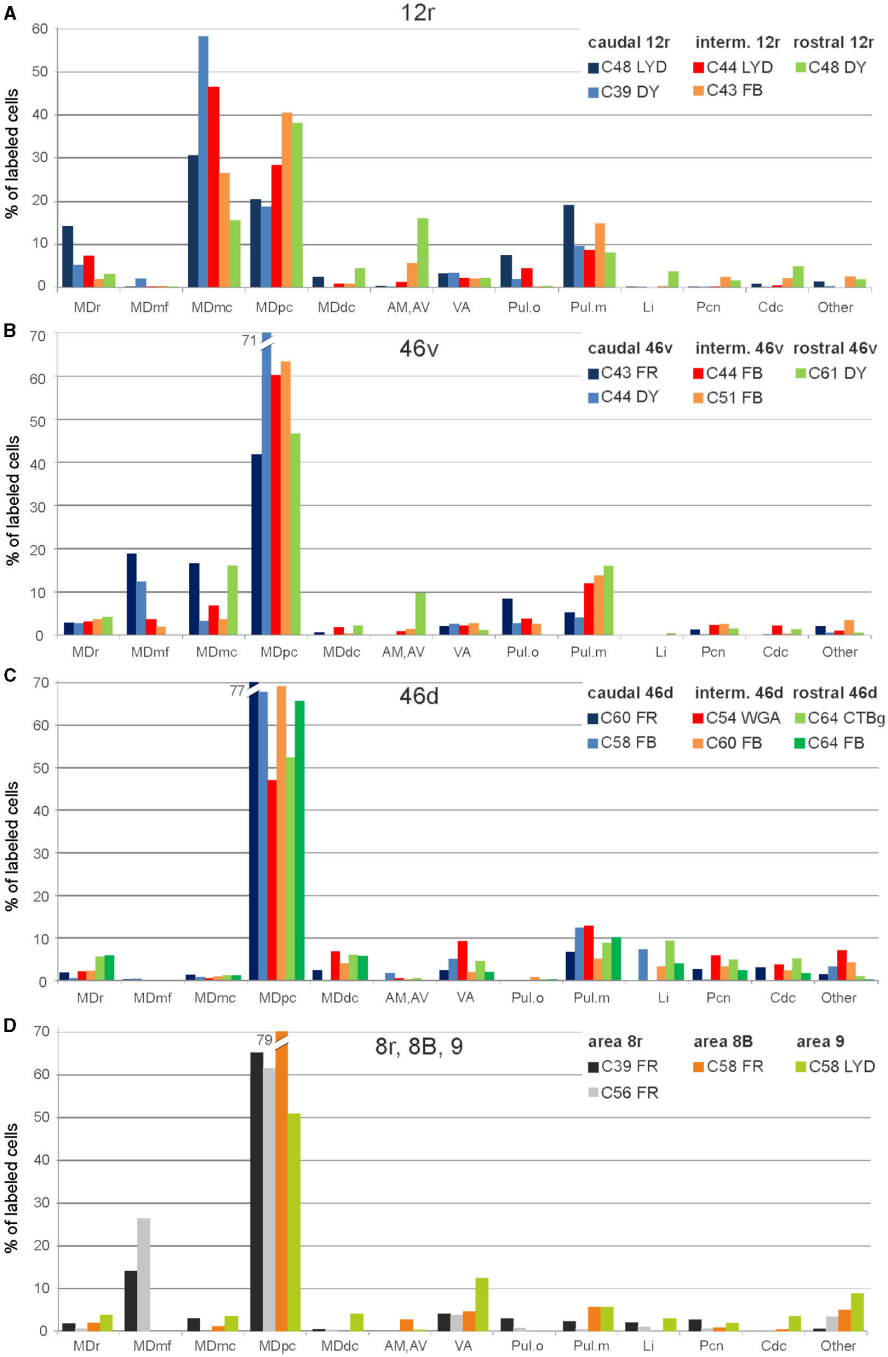
Percent distribution of the retrogradely labeled cells in the various thalamic nuclei observed after tracer injections in areas 12r **(A)**, 46v **(B)**, 46d **(C)**, and 8r, 8B, and 9 **(D)**. AM, nucleus anterior medialis; AV, nucleus anterior ventralis; Cdc, nucleus centralis densocellularis; Li, nucleus limitans; MDdc, nucleus medialis dorsalis, pars densocellularis; MDmc, nucleus medialis dorsalis, pars magnocellularis; MDmf, nucleus medialis dorsalis, pars multiformis; MDpc, nucleus medialis dorsalis, pars parvicellularis; MDr, nucleus medialis dorsalis, pars rostralis; Pul.o, nucleus pulvinaris oralis; Pul.m, nucleus pulvinaris medialis; Pcn, nucleus paracentralis; VA, nucleus ventralis anterior.

**Figure 3:**
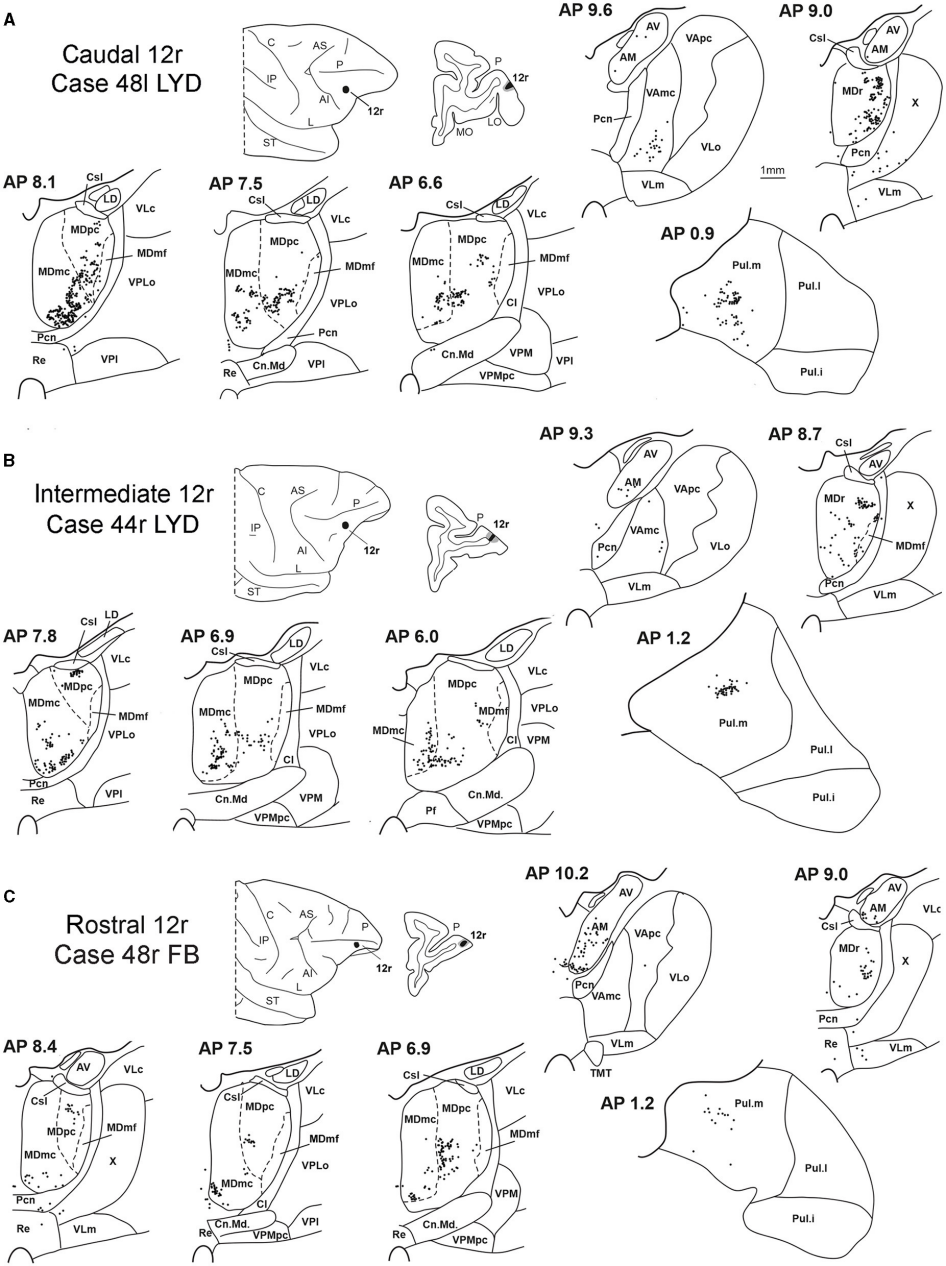
Distribution of retrogradely labeled thalamic neurons (represented by black dots) observed after the tracer injections in caudal, intermediate, and rostral area 12r, in Case 48l LYD **(A)**, Case 44r LYD **(B)**, and Case 48r FB **(C)**, respectively. The labeling is shown in drawings of coronal sections through the thalamus in rostral-to-caudal order, selected at different AP levels according to the atlas of Olszewski (1952). For each case, the location of the injection site is shown on a dorsolateral view of the injected hemisphere and in a coronal section through the core (shown in black) and halo (shown in lighter gray). For the sake of comparison, in this and subsequent figures, all thalamic and cortical section drawings, all drawings of the injected hemispheres, and all 3D reconstructions of the MD are shown as right. Cl, nucleus centralis lateralis; Cn.Md, nucleus centrum medianum; Csl, nucleus centralis superior lateralis; IP, intraparietal sulcus; L, lateral sulcus; LD, nucleus lateralis dorsalis; LO, lateral orbital sulcus; MO, medial orbital sulcus; Pf: nucleus parafascicularis; Pul.i, nucleus pulvinaris inferior; Pul.l, nucleus pulvinaris lateralis; Re, nucleus reuniens; ST, superior temporal sulcus; TMT, mammillothalamic tract; VLm, nucleus ventralis lateralis, pars medialis; VLo, nucleus ventralis lateralis, pars oralis; VAmc, nucleus ventralis anterior, pars magnocellularis; VApc, nucleus ventralis anterior, pars parvocellularis; VLc, nucleus ventralis lateralis, pars caudalis; VLo, nucleus ventralis lateralis, part oralis; VPLo, nucleus ventralis posterior lateralis, pars oralis; VPM, nucleus ventralis posterior medialis; VPMpc, nucleus ventral posterior medialis, pars parvocellularis; X, area X. Other abbreviations are shown in Figures 1, 2.

**Figure 4:**
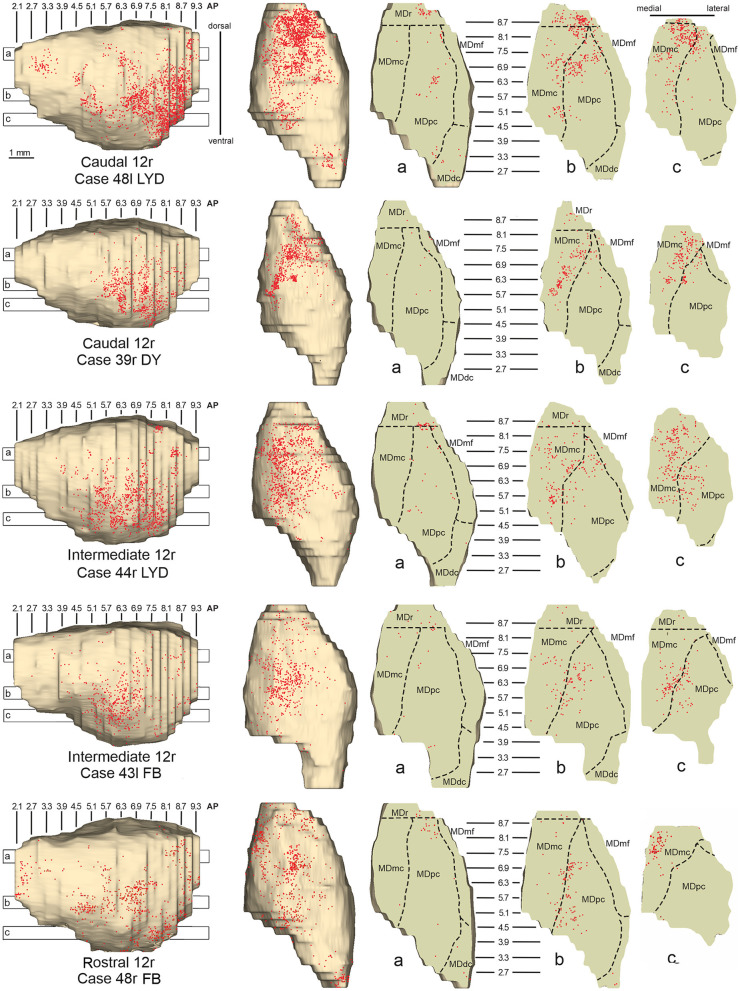
Distribution of retrogradely labeled neurons observed in the MD after the tracer injections in caudal (Cases 48l LYD and 39r DY, **upper part**), intermediate (Cases 44r LYD and 43l FB, **middle part**), and rostral (Case 48r FB, **lower part**) area 12r. For each case, the distribution of the labeling is shown, from left to right, in a lateral and a dorsal view of 3D reconstructions of the MD and in three horizontal sections 500 μm-thick, re-sliced from the 3D reconstructions shown in a dorsal-to-ventral order from a to c. The level at which each horizontal section was taken is indicated by the bar with the corresponding letter in the 3D reconstruction of the MD. Each dot corresponds to a labeled neuron. Dashed lines mark the borders between MD subdivisions. Abbreviations are shown in Figure 2.

Five tracer injections were placed at different rostrocaudal levels of area 12r (Figure 1). In all these cases, the labeling in the MD was located ventral and was characterized by a relatively high contribution of the MDmc, ranging from 16 to 58% of the total thalamic labeling (Figure 2). The distribution of the labeling varied according to the location of the injection site in area 12r. After the tracer injections in caudal area 12r (Cases 48l LYD and 39r DY), the labeling tended to be densest in the rostral part of the MD, especially in Case 48l LYD, which was the more posterior of the two tracer injections, involving the MDr and the rostral part of MDmc and MDpc up to approximately AP level 5.7 (Figures 3A, 4 upper part, Supplementary Figure 1A). After the tracer injections in intermediate area 12r (Cases 44l LYD and 43l FB), the MD labeled sector tended to overlap with the projection zone to caudal 12r, but the densest labeling tended to be located more caudal, especially in Case 43l FB (Figures 3B, 4 middle part, Supplementary Figure 1B). Finally, after the tracer injection in rostral 12r (Case 48r FB), the labeling was stronger in the MDpc than in MDmc (Figure 2) and was sparser in the rostrocaudal direction, involving both the MD zones labeled after the tracer injections in the caudal and intermediate 12r (Figures 3C, 4 lower part) and weakly also the MDdc.

Outside the MD nuclear complex, the most densely labeled nucleus was Pul.m (Figure 2), where the labeling tended to be more concentrated in a roughly central portion, at approximately AP 0.9–1.2 (Figure 3, Supplementary Figure 1). Clusters of labeled cells were also observed in the Pul.o after the tracer injections in caudal (both cases) and intermediate (Case 44r LYD) 12r, and in VAmc (Figure 3, Supplementary Figure 1) in all cases. Finally, in Case 48r FB (rostral 12r injection), there were approximately 11 and 9% of the labeled cells in AM and Limitans, respectively. Moderate labeling in AM was also observed in Case 43l FB (intermediate 12r injection).

### 3.2. Thalamic projections to area 46v

**Figure 5:**
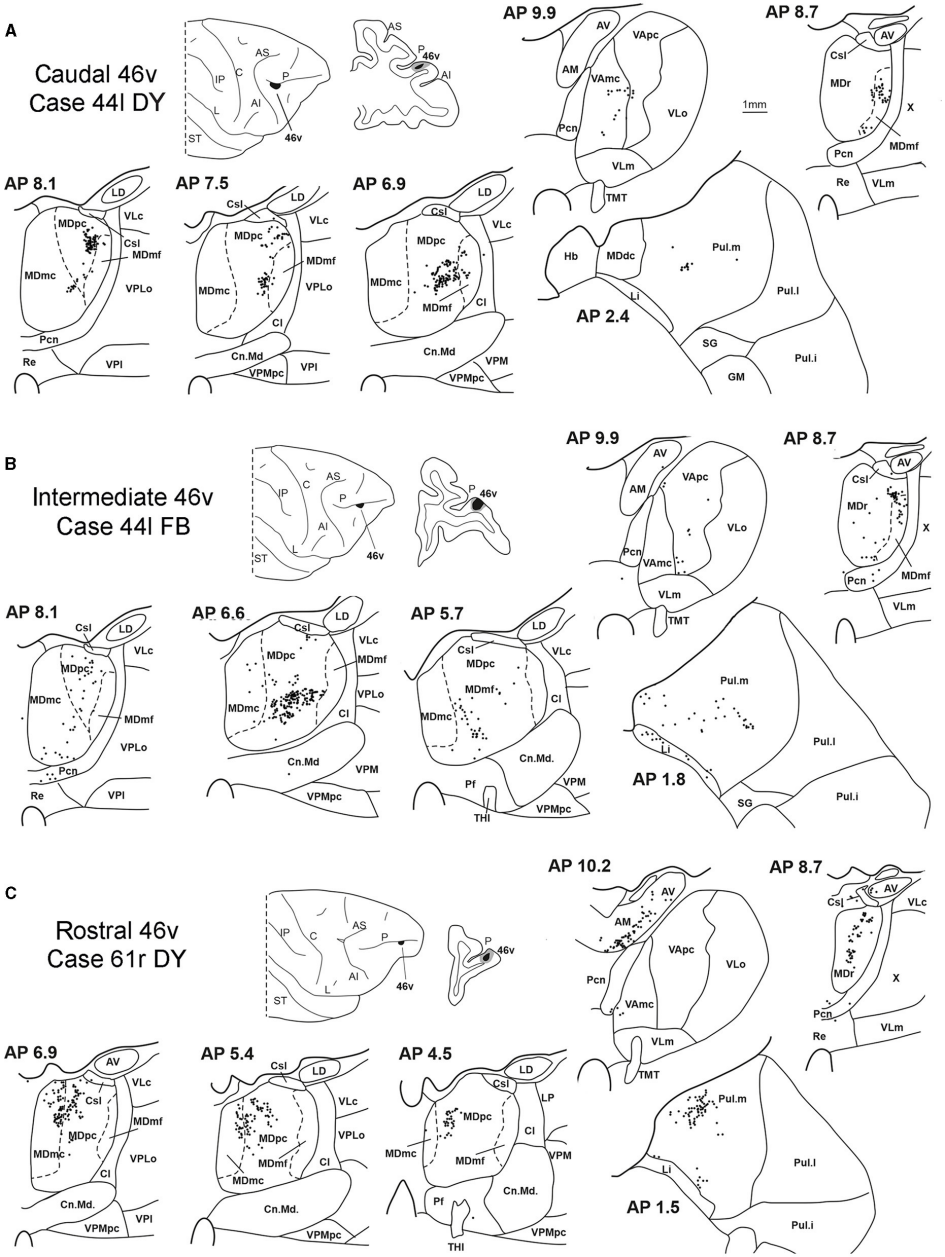
Distribution of retrogradely labeled thalamic neurons observed after the tracer injections in the caudal, intermediate, and rostral area 46v, in Case 44l DY **(A)**, Case 44l FB **(B)**, and Case 61r DY **(C)**, respectively. Format as in Figure 3. GM, nucleus geniculatus medialis; SG, nucleus suprageniculatus; THI, habenulo-interpeduncular tract. Other abbreviations are shown in Figures 1–3.

**Figure 6:**
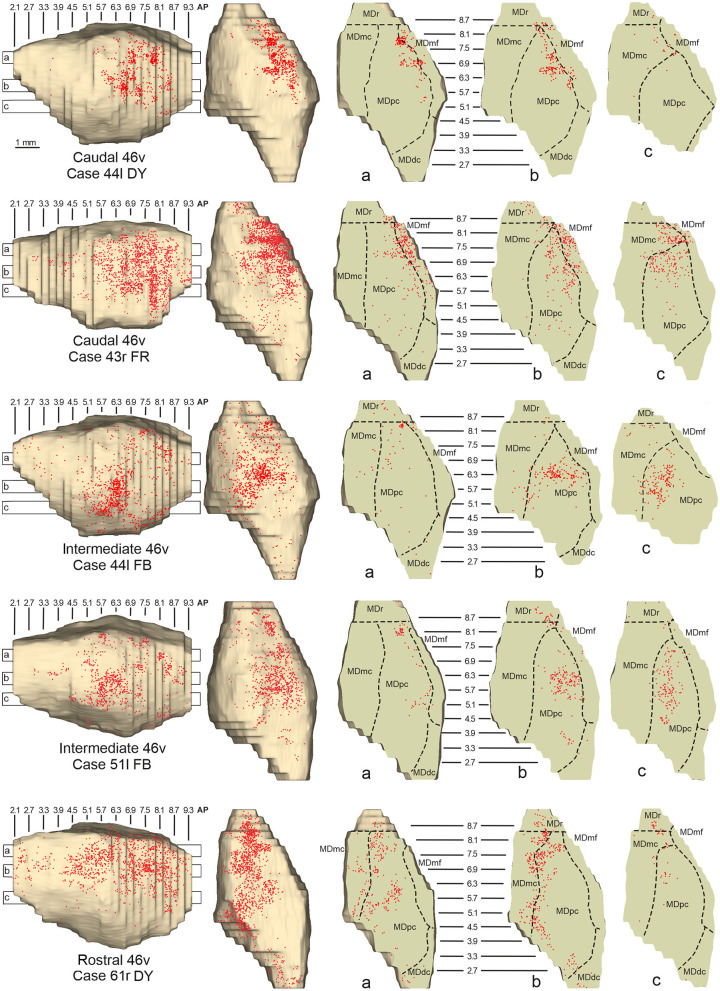
Distribution of retrogradely labeled neurons observed in the MD after the tracer injections in caudal (Cases 44l DY and 43r FR, **upper part**), intermediate (Cases 44l FB and 51l FB, **middle part**), and rostral (Case 61r DY, **lower part**) area 46v. Format as in Figure 4. Abbreviations are shown in Figure 2.

Five tracer injections were placed at different rostrocaudal levels of area 46v (Figure 1). Different from what was observed for area 12r, in all the cases of tracer injections in area 46v, the labeling was by far the densest in MDpc (Figure 2). As for area 12r, the distribution of the labeling in the MD varied according to the position of the injection site. After the tracer injections in caudal 46v (Cases 44l DY and 43r FR), the labeling was located laterally in the rostro-dorsal part of the MD, being densest between AP 8.1 and 6.3, robustly involving MDmf, and extending into the adjacent part of MDpc (Figures 5A, 6 upper part, Supplementary Figure 2A). In Case 43r FR, there was robust labeling also in rostral MDmc. After the tracer injections in intermediate area 46v, the labeling tended to be located more caudal and ventral, at about the same dorsoventral level of the MD projecting zone to area 12r. The labeling was densest from approximately AP levels 6.9 to 5.1, involving mostly the MDpc, tending to be located slightly more medial to, but likely overlapping with, the MD projecting zone to intermediate area 12r (Figures 5B, 6 middle part, Supplementary Figure 2B). The caudal shift of the labeling from the tracer injection in caudal 46v to that in intermediate 46v was particularly evident in Case 44l, in which DY and FB were injected in the caudal and intermediate 46v, respectively. As for area 12r, the labeling observed after the tracer injection in rostral 46v (Case 61r DY) widely extended in the rostrocaudal direction, in the dorsal and central parts of MDpc, densely involving also the adjacent part of the MDmc, thus likely largely segregated from the MD projecting zone to intermediate and caudal 46v (Figures 5C, 6, lower part).

Outside the MD nuclear complex, the most densely labeled nucleus was Pul.m, especially for intermediate and rostral 46v (Figures 2, 5, Supplementary Figure 2). Within this nucleus, the labeling tended to be more concentrated in a roughly central portion, except for Case 61r DY (rostral 46v injection), in which it tended to be located dorsal, at approximately AP 1.5–2.4. After the tracer injection in rostral 46v (Case 61r DY), relatively robust labeling was also located in AM.

### 3.3. Thalamic projections to area 46d

Six tracer injections were placed at different rostrocaudal levels of area 46d (Figure 1).

**Figure 7:**
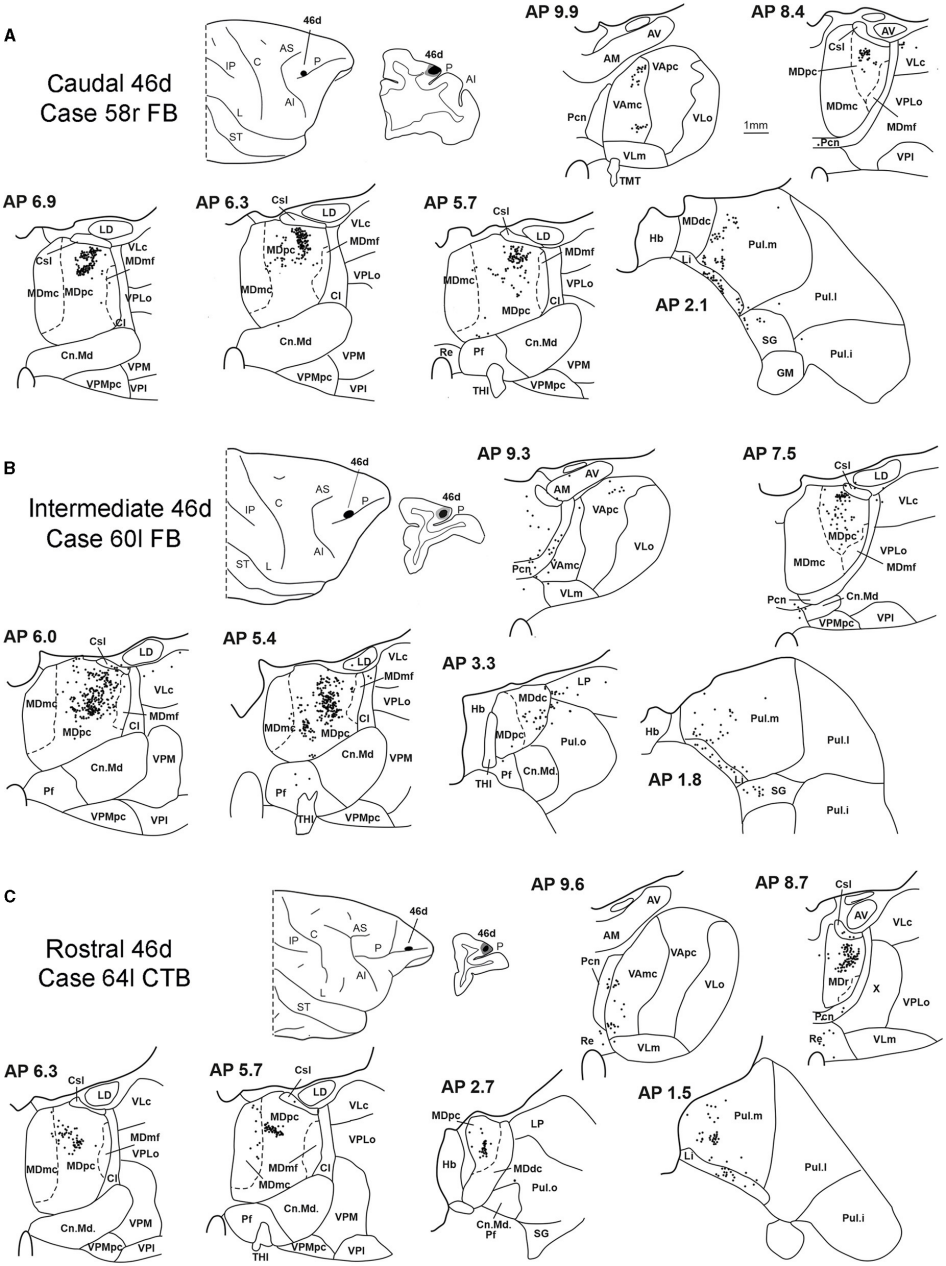
Distribution of retrogradely labeled thalamic neurons observed after the tracer injections in caudal, intermediate, and rostral area 46d, in Case 58r FB **(A)**, Case 60l FB **(B)**, and Case 64l CTB **(C)**, respectively. Format as in Figure 3. Other abbreviations are shown in Figures 1–3, 5.

**Figure 8:**
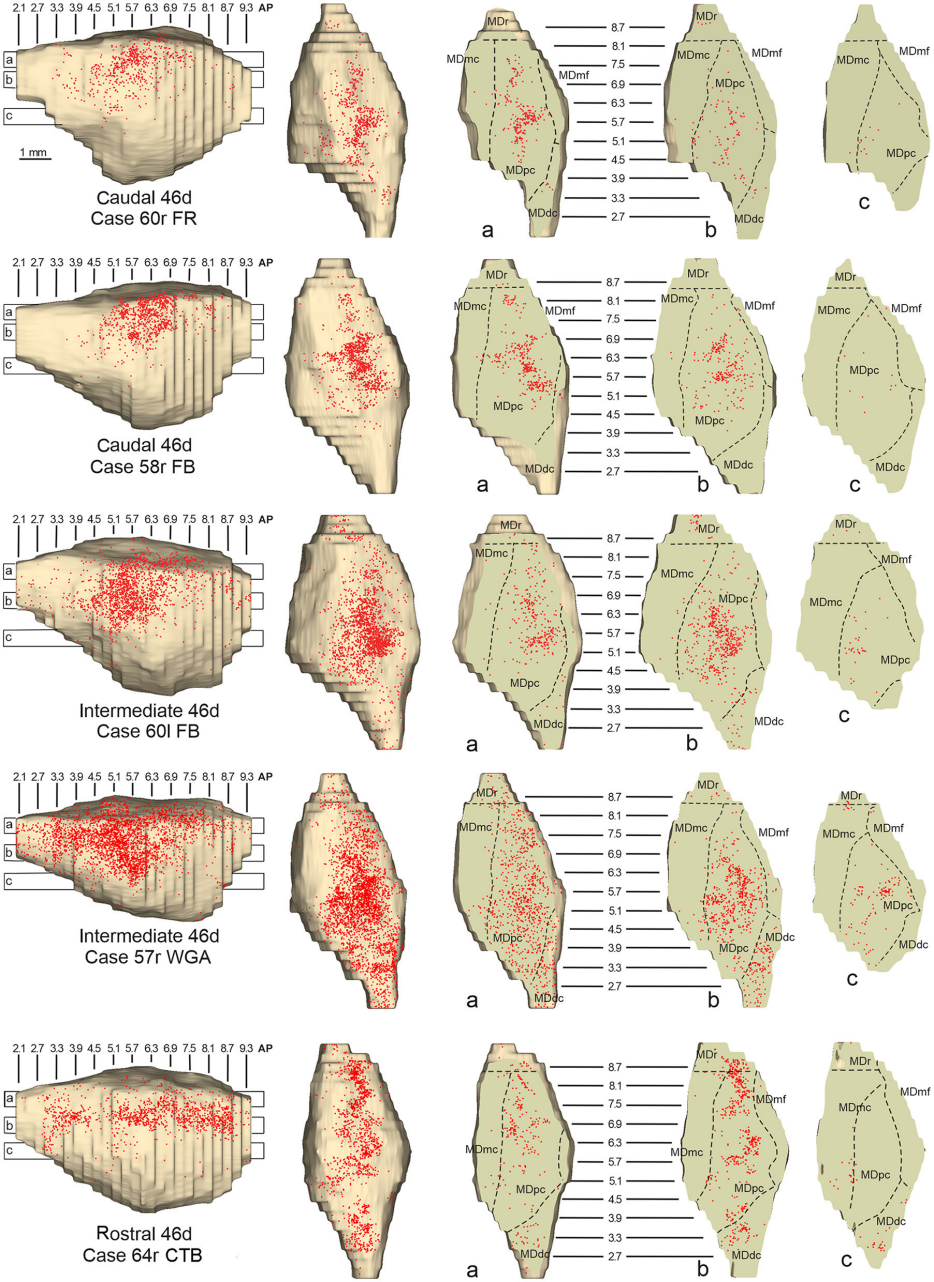
Distribution of retrogradely labeled neurons observed in the MD after the tracer injections in caudal (Cases 60r FR and 58r FB, **upper part**), intermediate (Cases 60l FB and 57r WGA, **middle part**), and rostral (Case 64r CTB, **lower part**) area 46d. Format as in Figure 4. Abbreviations are shown in Figure 2.

In all these cases, the labeling was located by far most in the MDpc (Figure 2), in the middle of its dorsal part. As observed for areas 12r and 46v, there was a tendency toward a shift in the rostrocaudal direction in the distribution of the labeled cells after the tracer injections in the caudal and in the intermediate 46d. In Cases 58r FB and 60r FR (caudal 46d injections), the labeling was densest between AP levels 6.9 and 5.1 (Figures 7A, 8 upper part, Supplementary Figure 3A). In Cases 60l FB and 57r WGA (intermediate 46d injections), the labeled zones extended more caudal, especially in Case 57r WGA, though largely overlapping with those projecting to caudal 46d (Figures 7B, 8 middle part, Supplementary Figure 3B). Furthermore, and similar to what was observed for rostral 12r and rostral 46v, after the tracer injections in Cases 64l CTB and 64r FB (rostral 46d injections), the labeled MD zone extended much more in the rostrocaudal direction, involving rostrally MDr and caudally MDdc, and largely overlapping with the projection zones to caudal and intermediate 46d (Figures 7C, 8 lower part, Supplementary Figures 3C, 4). This labeled zone appeared to be slightly more lateral but also overlapped with the zone labeled after the tracer injection in rostral 46v.

Outside the MD nuclear complex, the most densely labeled nucleus was Pul.m (Figure 2), where labeled cells tended to be sparsely distributed at approximately AP level 2.1–1.5, more medial than those observed after the tracer injections in areas 12r and 46v (Figure 7, Supplementary Figure 3). Weaker labeling was observed also in Li, especially after rostral 46d tracer injections, and in VAmc.

### 3.4. Thalamic projections to area 8r

**Figure 9:**
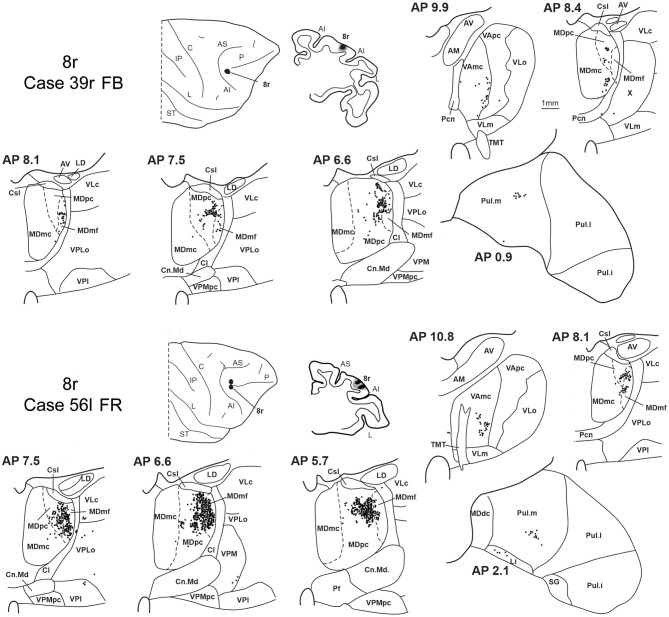
Distribution of retrogradely labeled thalamic neurons observed after the tracer injections in area 8r in Case 39r FB **(upper part)** and Case 56l FR **(lower part)**. Format as in Figure 3. Abbreviations are shown in Figures 1–3.

**Figure 10:**
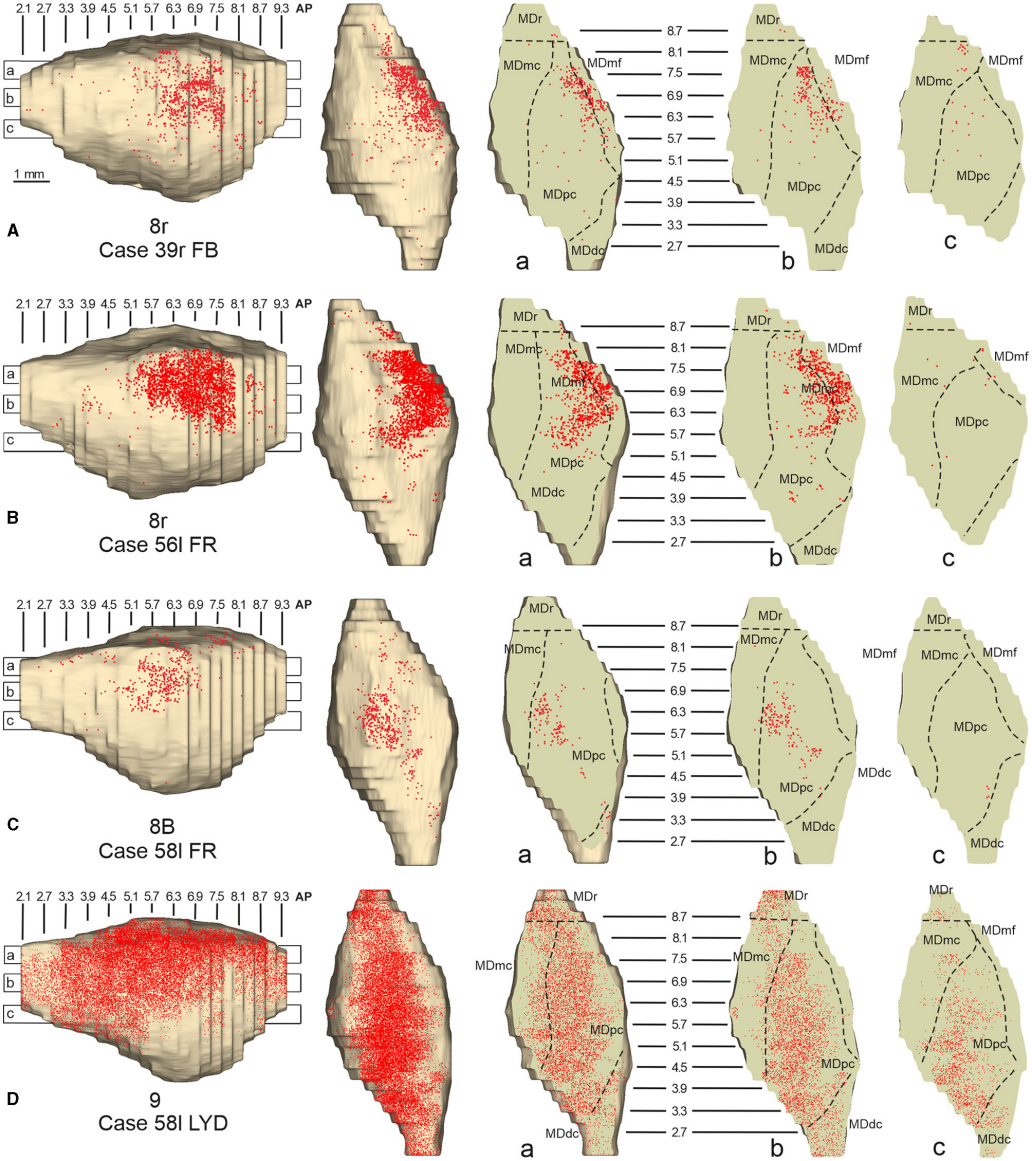
Distribution of retrogradely labeled neurons observed in the MD after the tracer injections in area 8r in Cases 39r FB **(A)** and 56l FR **(B)**, in area 8B in Case 58l FR **(C)**, and in area 9 in Case 58l LYD **(D)**. Format as in Figure 4. Abbreviations are shown in Figure 2.

Two tracer injections were placed in the prearcuate area 8r (Figure 1). In both cases, the labeling in the MD was confined to the lateral part of a dorsal and mid-rostral sector, densely involving the lateral most part of MDpc and MDmf (Figures 9, 10A, B), as expected from an area involved in the frontal oculomotor domain (Borra and Luppino, 2021), and largely overlapping with the MD sector labeled after the tracer injections in caudal 46v. Outside the MD nuclear complex, a few clusters of labeled cells were observed in VAmc, Pul.o, and Pul.m (Figure 9).

### 3.5. Thalamic projections to areas 8B and 9

**Figure 11:**
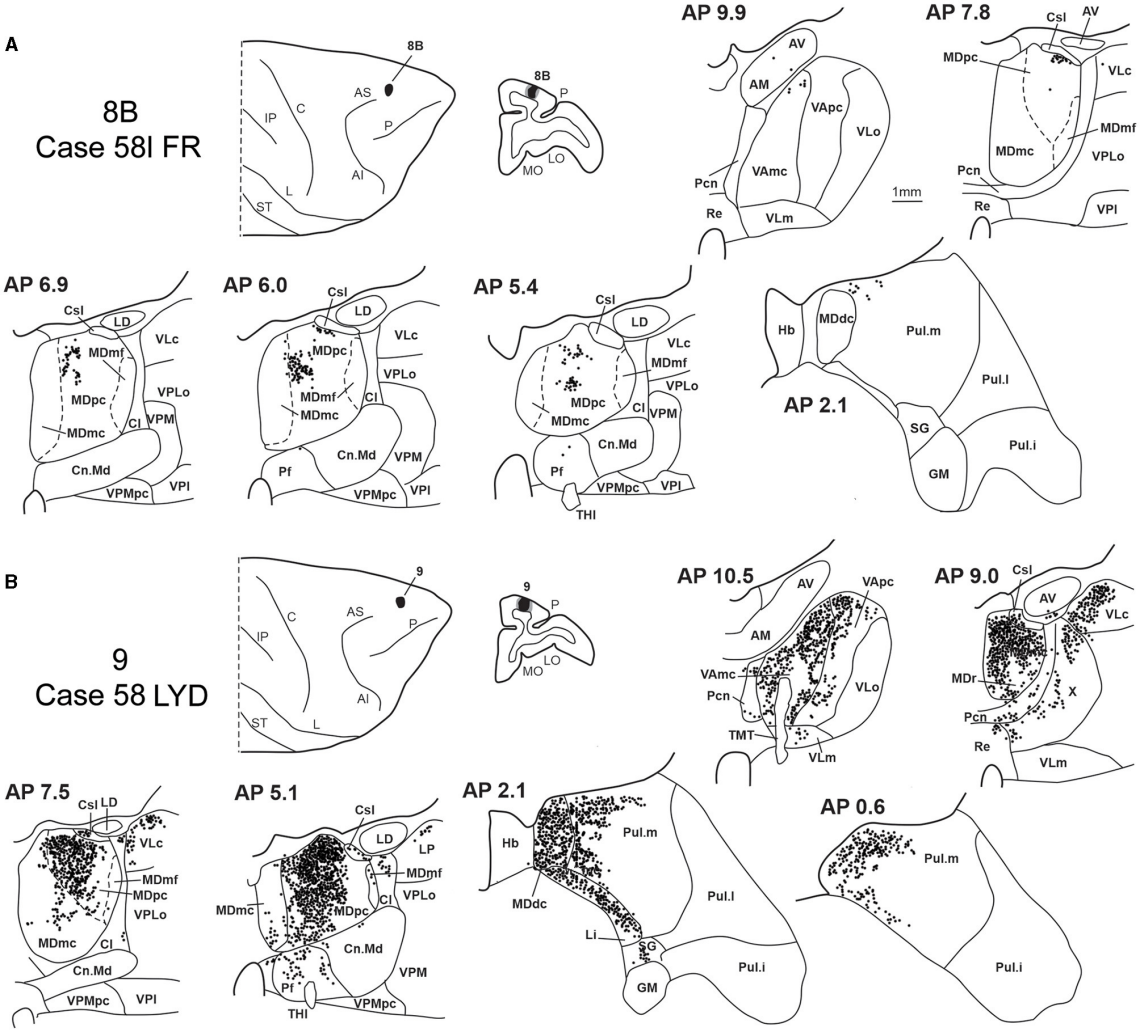
Distribution of retrogradely labeled thalamic neurons observed after the tracer injections in area 8B in Case 58l FR **(A)** and area 9 in Case 58l LYD **(B)**. Format as in Figure 3. Abbreviations are shown in Figures 1–3, 5.

Two tracer injections were placed in Case 58l in the dorsolateral convexity cortex dorsal to area 46d, one more caudal and one more rostral, in areas 8B and 9, respectively (Figure 1). In Case 58l FR (area 8B injection), the labeling was mostly confined to a MDpc zone located dorsal and medial, close to the border with MDmc at approximately AP levels 6.9–5.1 (Figures 2, 10C, 11A). Outside the MD nuclear complex, a few clusters of labeled cells were located dorsally in Pul.m at approximately AP level 2.1.

In Case 58l LYD, the thalamic labeling was particularly rich, densely involving the MD, mostly the dorsal part, along almost the entire rostrocaudal extent, including MDr, MDpc, and MDdc (Figures 2, 10D, 11B). This labeled zone appears to overlap at least partially with the MDpc zones projecting to areas 8B, 46d, and rostral 46v. Outside the MD nuclear complex, moderate labeling ranging from approximately 3–6% of the total thalamic labeling was found in the nucleus ventralis anterior, pars parvocellularis, VAmc, nucleus ventralis lateralis, pars caudalis, Pul.m, and limitans. Weaker labeling was observed in area X and in Pcn and Pf.

## 4. Discussion

The present study aimed to obtain a fine-grained view of the topography of the thalamic projections to the LPF in light of the data showing in this prefrontal region dorsolateral and rostrocaudal gradients of cortical connectivity.

Based on a relatively high number of tracer injections placed at different rostrocaudal and dorsoventral levels in the LPF, the present data extend previous studies in providing a more detailed and innovative view of the topography of the MD-LPF projections and illuminating aspects of the topography and divergence of these projections that have not been reported in previous studies.

### 4.1. Thalamic projections to the LPF

While Le Gros Clark (1932) identified the close association between the MD and the prefrontal cortex, and Rose and Woolsey (1948) proposed that the prefrontal cortex could be defined by its projections from MD, numerous other studies have shown MD connections to other cortical areas too, such as frontal motor and cingulate areas (e.g., Kievit and Kuypers, 1977; Schell and Strick, 1984; Goldman-Rakic and Porrino, 1985; Vogt et al., 1987; Giguere and Goldman-Rakic, 1988; Matelli et al., 1989; Darian-Smith et al., 1990; Matelli and Luppino, 1996; Rouiller et al., 1999; Hatanaka et al., 2003). Furthermore, several studies have shown connections between prefrontal areas with thalamic nuclei other than MD (e.g., Kievit and Kuypers, 1977; Goldman-Rakic and Porrino, 1985; Barbas et al., 1991; Romanski et al., 1997; Xiao et al., 2009). Nevertheless, the MD is by far the major source of thalamic input to the prefrontal cortex, contributing, for example, to the present study, with 48–89% of the thalamic neurons projecting to LPF areas.

Several studies (Pribram et al., 1953; Kievit and Kuypers, 1977; Goldman-Rakic and Porrino, 1985; Barbas et al., 1991; Siwek and Pandya, 1991; Ray and Price, 1993; Erickson and Lewis, 2004; Phillips et al., 2019) have already described a topographic organization of the MD-LPF projections, and several models have been proposed, which, however, failed to converge. In most of these models, mapping onto MD reflects the position in the prefrontal cortex of the target cortical area. Pribram et al. (1953), based on large cortical lesions, suggested that the circumference of the prefrontal cortex is mapped from medial to lateral onto the MD, with dorsolateral, ventrolateral, and orbital cortex represented in lateral, central, and medial MD, respectively, and rostral-to-caudal cortical areas represented from dorsal-to-ventral in the MD. Different from Pribram et al. (1953), based on very large cortical tracer injections, they proposed that rostral-to-caudal prefrontal parts are mapped onto medial-to-lateral and not dorsal-to-ventral bands in MD. Goldman-Rakic and Porrino (1985), again based on very large tracer injections in the prefrontal cortex, put forward a model in which the circumference of the prefrontal cortex is mapped onto the circumference of the MD, but rostral-to-caudal gradients were not taken into account in this study. Based on large tracer injections in the MD, Erickson and Lewis (2004) described three MD sectors associated with a multiplicity of areas located in different cortical regions: a ventrolateral one, connected to caudal prefrontal and dorsal and ventral premotor areas, a caudoventral one, connected to dorsomedial areas including the pre-SMA and area 24, and an anterodorsal one, connected to more rostral prefrontal areas 9, 46, 12, and 10. Finally, Phillips et al. (2019) using a diffusion magnetic resonance imaging approach showed an ordered topographic gradient of MD-prefrontal connections in which the anteromedial part of MD is connected mostly with ventromedial and orbital prefrontal regions, while most posterolateral MD is connected preferentially with posterolateral prefrontal regions, and intermediate prefrontal regions are connected with the zone in between. This study, different from the previous ones, suggested that rostral-to-caudal prefrontal areas are mapped onto the rostrocaudal dimension in the MD without any evident topographic organization in the ventral-to-dorsal dimensions, as projection zones to either dorsal and ventral prefrontal areas were found at similar dorsoventral MD levels. In other models, mapping onto the MD reflects the degree of architectonic differentiation of prefrontal areas. This is the case of the studies of Barbas et al. (1991) and Siwek and Pandya (1991), based on tracer injections in the prefrontal cortex and in the MD, respectively, which agreed on a rather general model in which architectonically less differentiated areas, such as orbitofrontal areas, have a strong relationship with medial MD, whereas more differentiated ones, such as caudal prefrontal areas, have a strong relationship with lateral MD.

All these studies, based on: (i) relatively large lesions or tracer injections in the prefrontal cortex or in MD, (ii) a rather limited number of tracer injections placed in far apart prefrontal areas, or (iii) tractographic magnetic resonance approaches, could not provide fine-grained descriptions of the MD-LPF connectivity.

**Figure 12:**
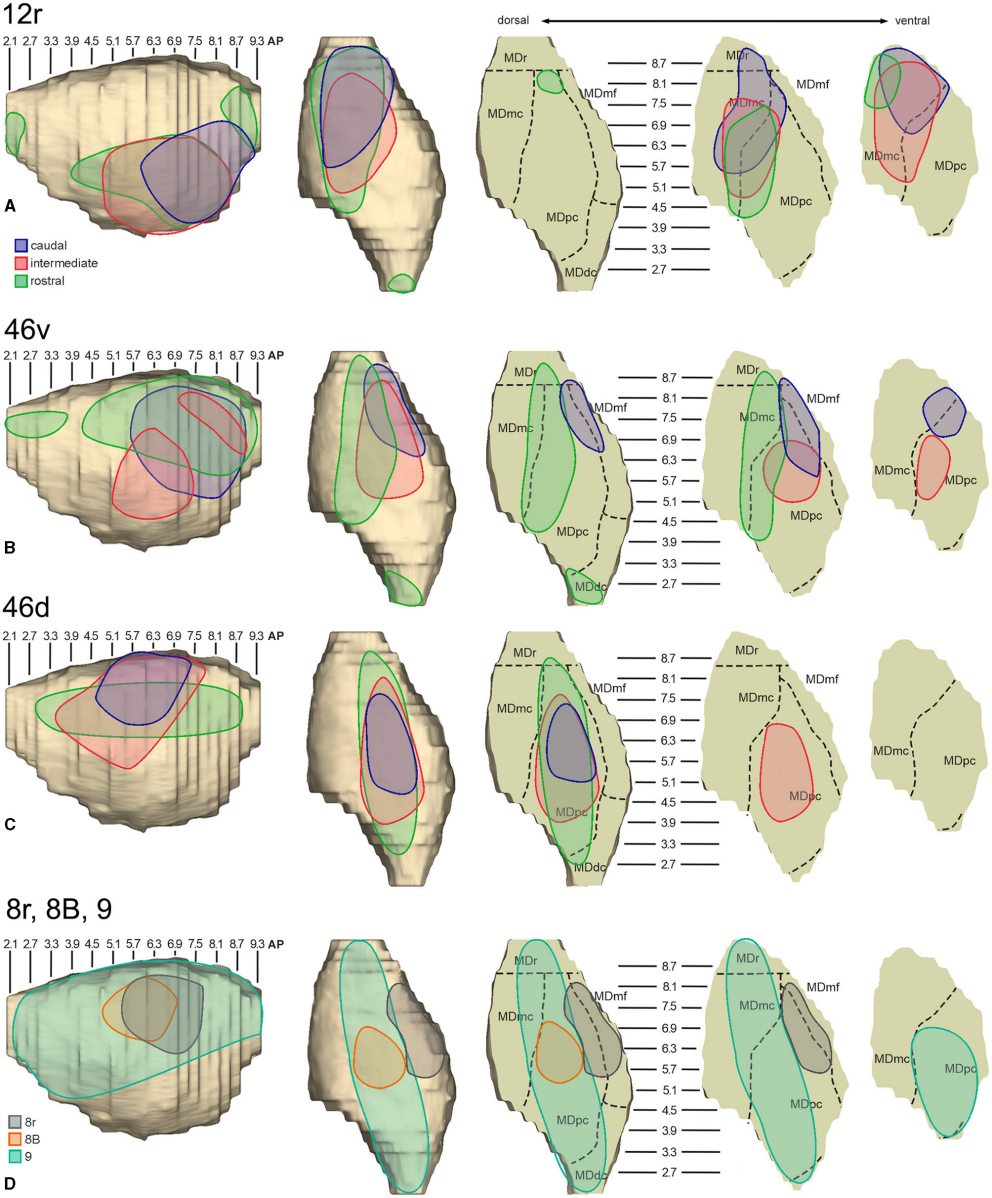
Composite view of the MD labeled sectors (superimposed on the 3D reconstruction of the MD of Case 44r) observed after tracer injections at different rostrocaudal levels in areas 12r **(A)**, 46v **(B)**, and 46d **(C)**, and in areas 8r, 8B, and 9 **(D)**.

In general, the present data summarized in Figure 12 agree with previous studies showing that most of the LPF is a target of thalamic projections originating primarily from MDpc (Goldman-Rakic and Porrino, 1985; Barbas et al., 1991; Siwek and Pandya, 1991; Ray and Price, 1993). Furthermore, they extend observations from Barbas et al. (1991) and Ray and Price (1993), based on one tracer injection placed in area 12r, showing that the entire extent of this area is a target of strong projections from MDmc, which also involve, at a lesser extent, rostral 46v. Finally, the data extend observations from Barbas et al. (1991), based on one tracer injection in caudal 46v, showing that the MD projections to both areas, caudal 46v and 8r, originate most densely from a rostral and lateral MD zone, including MDmf. This MD zone shows a large overlap with the MD projection zone to prearcuate oculomotor areas 8-FEF and 45B (Huerta et al., 1986; Barbas et al., 1991; Contini et al., 2010).

As for the topography of the MD-LPF projections, the present data first show that there are several exceptions to circumferential models in which dorsal-to-lateral LPF positions are mapped onto dorsal-to-ventral MD zones (e.g., Goldman-Rakic and Porrino, 1985; Barbas et al., 1991; Siwek and Pandya, 1991). Specifically, the MD projecting zone to intermediate 46v was slightly shifted medially but located at the same dorsoventral level as that projecting to the caudal and intermediate 12r. Furthermore, the MD zones projecting to the various 46d sectors largely overlapped with those projecting to the more dorsal areas 8B and 9. Finally, labeled cells projecting to rostral 46v were located at the same dorsoventral level as those projecting to 46d, 8B, and 9.

The mapping onto the MD of rostral-to-caudal LPF zones observed in the present study represents the major difference between the present and previous data. Indeed, we found that the MD zones projecting to the caudal part of 12r, 46v, and 46d tended to be located quite rostrally in the MD, as well as the more caudal prearcuate oculomotor areas (Contini et al., 2010). Furthermore, the MD zones projecting to the intermediate part of areas 12r, 46v, and 46d tended to shift caudally with respect to those projecting to the caudal part of the same areas. Finally, the MD zones projecting to the rostral part of areas 12r, 46v, and 46d tended to be much more extensive in the rostrocaudal direction covering the AP levels of those projecting to the caudal and intermediate parts of the same area. Accordingly, the present data do not support models in which rostral-to-caudal LPF positions are mapped onto dorsal-to-ventral (Pribram et al., 1953), medial-to-lateral strips (Kievit and Kuypers, 1977), or in which more caudal and more rostral LPF areas are mapped onto more caudal and more rostral MD zones, respectively (Phillips et al., 2019). Thus, the present data suggest that the topography of the MD-LPF projections does not obey simple topological rules.

Several studies based on tracer injections in the MD observed label in multiple, spatially segregated patches, involving different areas, in some cases extensively involving large cortical regions of the frontal cortex (Russchen et al., 1987; Giguere and Goldman-Rakic, 1988; Yeterian and Pandya, 1994; McFarland and Haber, 2002; Erickson and Lewis, 2004; Xiao et al., 2009). It has then been suggested that a large degree of divergence is consistent with the role of MD in coordinating communication across large prefrontal regions. The present data provide evidence for divergento projections to different groups of LPF areas from three specific MD zones. One rostrolateral MD zone involving MDr, MDmf, the adjacent part of MDpc, and MDmc appears to be a common source of projections to caudal 12r, 46v, and 8r, as well as to areas 8-FEF and 45B (Contini et al., 2010). A further MD sector located more caudal and ventral occupies an MDpc zone, which appears to project to the entire extent of 12r, to intermediate 46v, and areas 45A and 45B (Contini et al., 2010), and a MDmc zone, which appears to project to the entire extent of area 12r, to areas 45A and 45B (Contini et al., 2010), and to orbitofrontal areas 11, 13, and 12o (Goldman-Rakic and Porrino, 1985; Barbas et al., 1991; Siwek and Pandya, 1991; Morecraft et al., 1992; Ray and Price, 1993). A third MD sector occupies a dorsal and middle zone of MDpc and appears to be a common source of projections to the entire extent of area 46d, areas 9 and 8B, and the rostral part of area 46v.

As for the projections to LPF from thalamic nuclei other than MD, the present data, in agreement with other studies (Goldman-Rakic and Porrino, 1985; Barbas et al., 1991; Romanski et al., 1997), showed moderate projections from Pul.m and relatively weak projections from VA to all injected areas. In Pul.m, as observed by Goldman-Rakic and Porrino (1985) and Romanski et al. (1997), the labeling showed a mediolateral topography with Pul.m sectors projecting to areas 46v and 12r located in a middle zone, lateral to one projecting to areas 46d, 8B, and 9, and medial to one projecting to areas 8r, 8-FEF, and 45B (Contini et al., 2010). Furthermore, the present data showed projections from AM to the rostral part of areas 12r and 46v. Projections from AM have been observed by Goldman-Rakic and Porrino (1985) after very large injections in ventral LPF.

### 4.2. Functional considerations

MD and Pul.m, which are the major sources of thalamic projections to LPF, both are higher order nuclei (Guillery, 1995), i.e., nuclei in which thalamocortical cells receive their driving input mostly from layer V cortical neurons and a modulatory input from layer VI cortical neurons. Higher-order thalamic relays also receive subcortical inputs that have either an excitatory or modulatory function.

In addition to LPF, MD and Pul.m are connected to cingulate, frontal motor, parietal, and temporal areas (e.g., Kievit and Kuypers, 1977; Schell and Strick, 1984; Goldman-Rakic and Porrino, 1985; Yeterian and Pandya, 1985; Matelli et al., 1989; Darian-Smith et al., 1990; Schmahmann and Pandya, 1990; Dum and Strick, 1993; Matelli and Luppino, 1996; Romanski et al., 1997; Rouiller et al., 1999; Gutierrez et al., 2000; Stepniewska, 2004; Morel et al., 2005; Cappe et al., 2007, 2009) with some overlap of the territories connected with different prefrontal areas or prefrontal and non-prefrontal areas.

Furthermore, cortical projections to these nuclei tend to be more extensive than thalamocortical projections, suggesting non-reciprocal connections. This connectional organization represents the neural substrate for relaying already processed cortical information onto other cortical areas, thus subserving trans-thalamic corticocortical transfer of neural signals, and is known as the replication principle (Shipp, 2003). In this context, modulatory inputs from cortical and subcortical structures could influence how and what driving signals get relayed to the cortex via this indirect trans-thalamic route (for reviews, see e.g., Mitchell, 2015; Perry et al., 2021; Phillips et al., 2021).

By virtue of its extensive connectivity with the prefrontal cortex, the MD appears to contribute to several aspects of cognitive control. In general, this nucleus could play an essential role in controlling the excitability of cortical neurons as well as flexibly routing information between cortical neurons according to context, supporting the transfer of information across the cortex during learning and decision-making (Mitchell, 2015; Phillips et al., 2021). Based on their differential patterns of cortical and subcortical connectivity, the various MD subdivisions appear to support different aspects of cognitive functions. Specifically, MDmc, which receives driving input from the amygdala and is connected mostly with medial and orbital prefrontal areas, is considered to be necessary for monitoring the outcome of behavioral responses based on recent choices (Perry et al., 2021), thus contributing to value-to-choice transformations (Phillips et al., 2021). MDpc, which receives a driving input from the cortex, appears to be involved in different aspects of executive functions, including working memory tasks in which neurons appear to code forthcoming motor information based on sensory input provided by the LPF (Funahashi, 2013). Furthermore, a recent study showed that MDpc neurons can be specialized for decision-making and response selection, whereas prefrontal neurons can be specialized in coding the contextual information on which the decision is based (DeNicola et al., 2020). Accordingly, the MDpc could contribute to rule-to-action transformations (Phillips et al., 2021). Finally, MDmf is a robust source of thalamic input to all the various oculomotor frontal areas, including area 8-FEF, 45B, and the supplementary eye field (Huerta et al., 1986; Stanton et al., 1988; Shook et al., 1991; Matelli and Luppino, 1996; Contini et al., 2010), and is a target of subcortical input from the superior colliculus and the substantia nigra pars reticulata (Harting et al., 1980; Ilinsky et al., 1985; Erickson et al., 2004). Neurons in this nucleus display presaccadic activity considered a corollary discharge originating from the superior colliculus and relayed to frontal oculomotor areas, which facilitates the re-mapping of retinotopic receptive fields in the cortex during eye movements (Sommer and Wurtz, 2004, 2008), thus contributing to motor-to-sensory transformations (Phillips et al., 2021).

Our data appear to be quite congruent with the notion that the MD is a neural substrate for trans-thalamic corticocortical transfer of neural signals between areas belonging to a specific functional domain. In this context, the rostrolateral MD sector involving MDr, MDmf, MDpc, and MDmc could represent a site for the exchange of sensory and motor signals between areas involved in the frontal oculomotor domain (Borra and Luppino, 2021) and for relaying trans-thalamic amygdalar and orbitofrontal information to caudal areas 12r and 46v, as well as to areas 45A and 45B (Contini et al., 2010). Thus, this rostrolateral MD sector could be more specifically involved in the executive control of oculomotor behavior. The present data provide further evidence for the involvement of caudal 46v and 8r in the frontal oculomotor domain, as suggested by data on their corticocortical and subcortical connectivity (Gerbella et al., 2013; Borra et al., 2015). Furthermore, in the more caudal and ventral MD sectors, the MDmc component, as for the more rostral sector, could contribute to value-to-action transformations, and the MDpc component to working memory of non-spatial information, decision-making, and action selection finalized to executive control of oculomotor and skeletomotor behavior in ventral LPF. Finally, the dorsal MD sector corresponding to a dorsal and middle portion of MDpc could mediate the transfer of information between dorsal LPF areas for visuospatial working memory (Levy and Goldman-Rakic, 2000; Funahashi, 2013) or higher-order aspects of cognitive control such as hierarchical representation of task events (Sigala et al., 2008), retrieval and integration of task-relevant information for action planning (Hoshi and Tanji, 2004; see also Tanji and Hoshi, 2008), and selection of abstract response strategies (Genovesio et al., 2005; Tsujimoto et al., 2011). Dorsal LPF areas are also targets of projections from the medial part of Pul.m, which could mediate the transfer of information originating from rostral temporal and cingulate areas and the amygdala (Romanski et al., 1997). In contrast, ventral LPF areas, as well as oculomotor frontal areas, are targets of projections from more central and lateral zones of Pul.m, which could mediate the transfer of information originating from posterior parietal, insular, superior, and inferior temporal areas, and the posterior cingulate cortex (Romanski et al., 1997). This trans-thalamic corticortical flow of information through Pul.m could contribute to sensory-to-choice transformations (Phillips et al., 2021).

The present data also show that the MD projection zone to the rostral part of LPF areas 12r, 46v, and 46d is quite extensive in the rostrocaudal direction and includes zones projecting to more caudal LPF and to premotor and cingulate motor areas (e.g., Matelli and Luppino, 1996; Erickson and Lewis, 2004; Morel et al., 2005; Cappe et al., 2009). Furthermore, the rostral part of areas 12r and 46v are targets of projections from AM, a subdivision of the anterior thalamic nuclei connected to anterior cingulate and medial and orbitofrontal areas, and the target of projections from hippocampal formation (subicular cortex and CA3), entorhinal, perirhinal and parahippocampal cortex, and the amygdala (see, e.g., Child and Benarroch, 2013; Phillips et al., 2021). Thus, AM could mediate the transfer of mnemonic and affective signals for higher-order aspects of cognitive functions. This connectivity pattern is congruent with proposed models of hierarchical organization of the frontal lobe in which, moving from caudal to rostral in the human prefrontal cortex, there is an increase in the levels of cognitive control through the processing of increasingly abstract representations (Koechlin and Summerfield, 2007; Badre and D'Esposito, 2009).

## Data availability statement

The raw data supporting the conclusions of this article will be made available by the authors, without undue reservation.

## Ethics statement

The animal study was approved by Veterinarian Animal Care and Use Committee of the University of Parma and Italian Ministry of Health. The study was conducted in accordance with the local legislation and institutional requirements.

## Author contributions

EB and GL designed and performed research and wrote the manuscript. EB, MR, and GL analyzed the data and edited the manuscript. All authors contributed to the article and approved the submitted version.
