# Supplementary material for: Gradients of thalamic connectivity in the macaque lateral prefrontal cortex
Source: Front Integr Neurosci. 2023 Oct 16;17:1239426. doi: 10.3389/fnint.2023.1239426 (PMC10613699; doi:10.3389/fnint.2023.1239426)
Supplement: Supplementary file 1 [file Data_Sheet_1.DOCX]

SUPPLEMENTARY MATERIAL

**Gradients of thalamic connectivity in the macaque lateral prefrontal cortex**

**Elena Borra, Marianna Rizzo, Giuseppe Luppino^*^**

Neuroscience Unit, Department of Medicine and Surgery, University of Parma, Parma, Italy

Correspondence: Giuseppe Luppino*

[giuseppe.luppino@unipr.it](mailto:giuseppe.luppino@unipr.it)

**
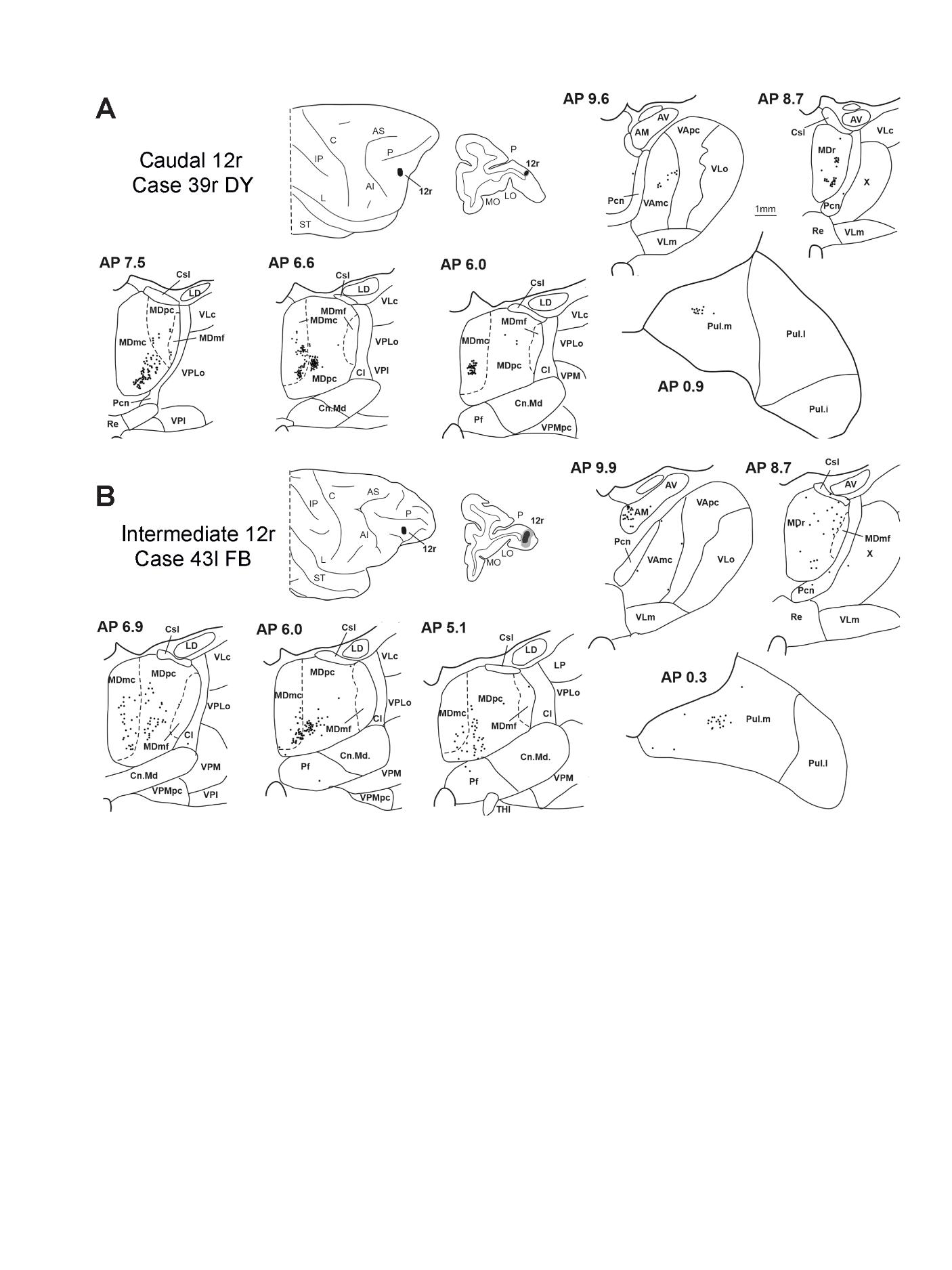
**

**Supplementary Figure 1.** Distribution of retrogradely labeled thalamic neurons observed after the tracer injections in caudal and intermediate area 12r in Case 39r DY (**A**) and Case 43l FB (**B**), respectively. Format as in Figure 3. Abbreviations as in Figures 1 to 3, and 5.


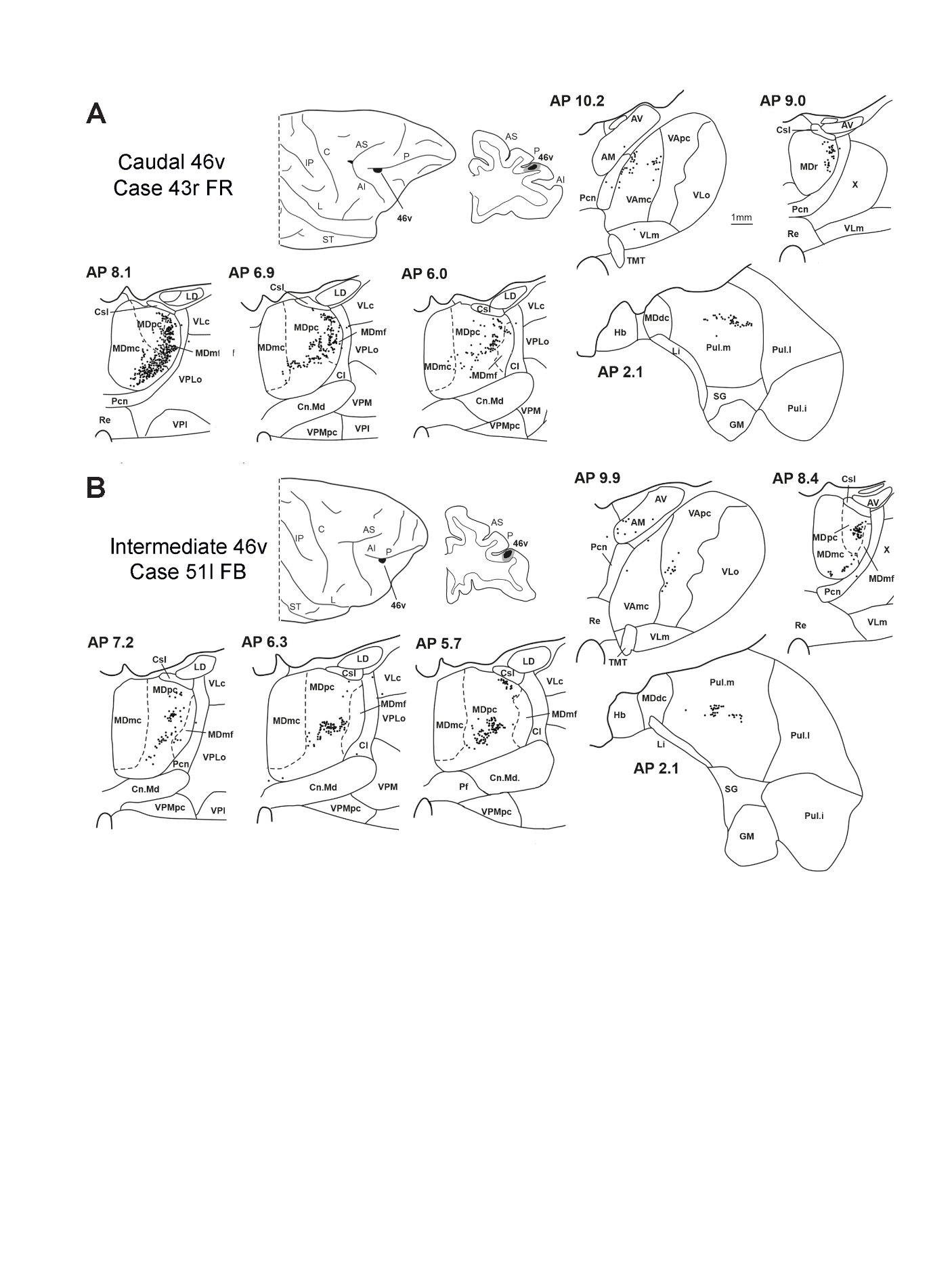


**Supplementary Figure 2.** Distribution of retrogradely labeled thalamic neurons observed after the tracer injections in caudal and intermediate area 46v in Case 43r FR (**A**) and Case 51l FB (**B**), respectively. Format as in Figure 3. Abbreviations as in Figures 1 to 3, and 5.


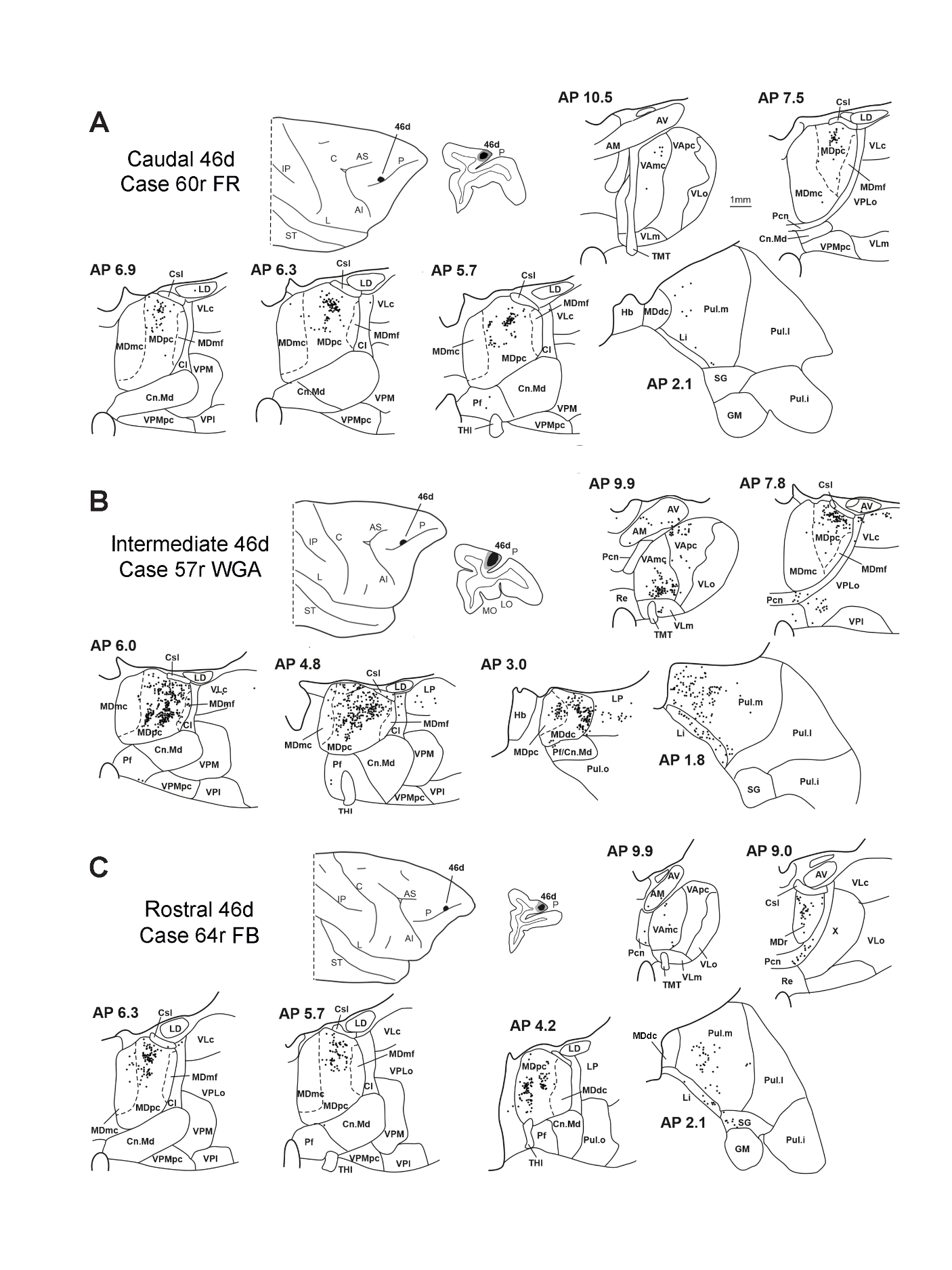


**Supplementary Figure 3.** Distribution of retrogradely labeled thalamic neurons observed after the tracer injections in caudal, intermediate, and rostral area 46d, in Case 60r FR (**A**), Case 57r WGA (**B**), and Case 64r DY (**C**), respectively. Format as in Figure 3. Abbreviations as in Figures 1 to 3.


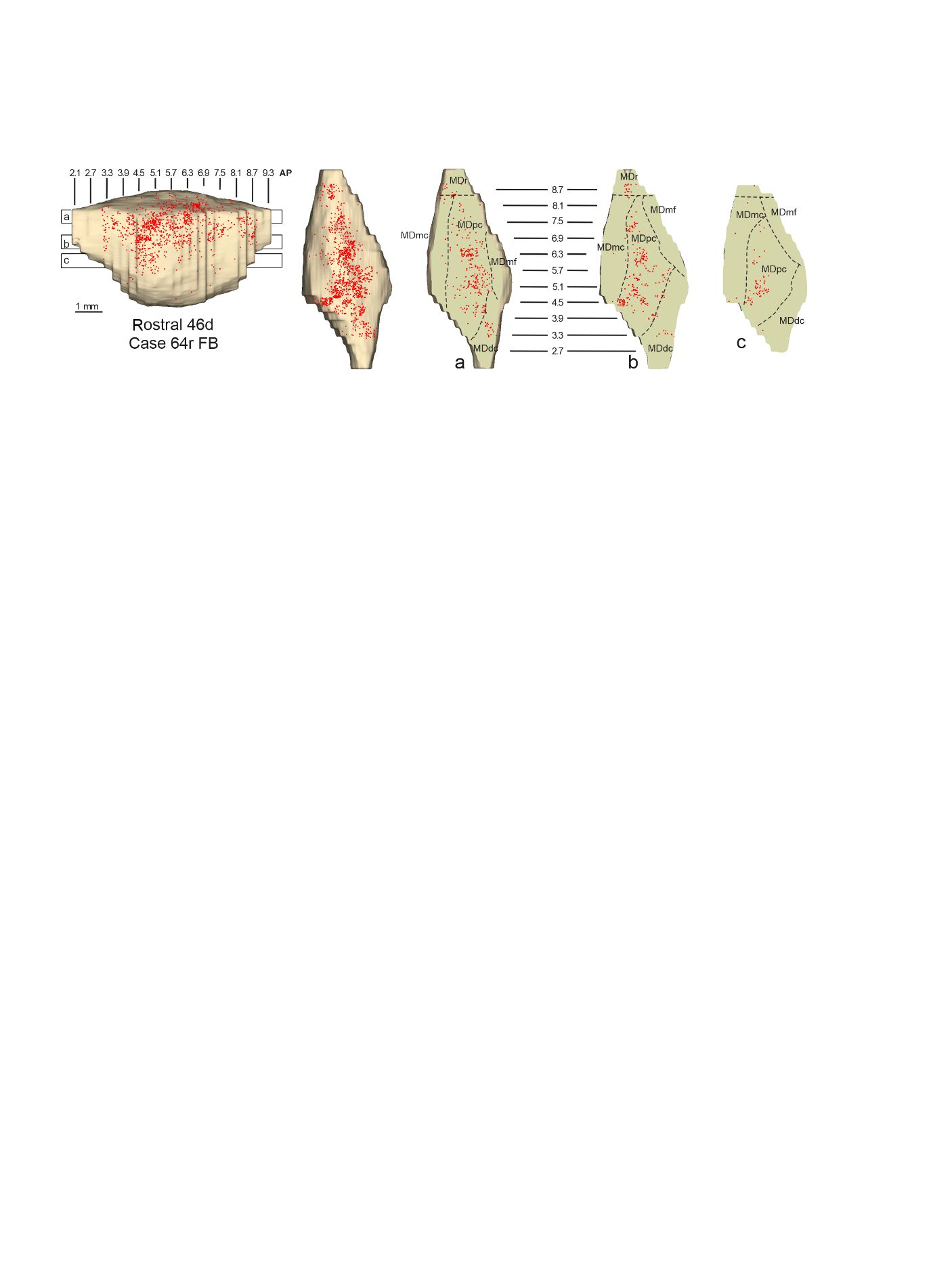


**Supplementary Figure 4.** Distribution of retrogradely labeled neurons observed in the MD after the tracer injection in area 46d in Case 64r FB. Format as in Figure 4. Abbreviations as in Figure 2.
